# Diagnostic performance of MRI in detecting prostate cancer in patients with prostate-specific antigen levels of 4–10 ng/mL: a systematic review and meta-analysis

**DOI:** 10.1186/s13244-024-01699-4

**Published:** 2024-06-18

**Authors:** Erjia Guo, Lili Xu, Daming Zhang, Jiahui Zhang, Xiaoxiao Zhang, Xin Bai, Li Chen, Qianyu Peng, Gumuyang Zhang, Zhengyu Jin, Hao Sun

**Affiliations:** 1 Department of Radiology, State Key Laboratory of Complex Severe and Rare Disease, Peking Union Medical College Hospital, Peking Union Medical College, Chinese Academy of Medical Sciences, Shuaifuyuan No.1, Wangfujing Street, Dongcheng District, Beijing, 100730 China; 2 National Center for Quality Control of Radiology, Shuaifuyuan No.1, Wangfujing Street, Dongcheng District, Beijing, 100730 China

**Keywords:** Prostate neoplasms, Prostate-specific antigen, Magnetic resonance imaging

## Abstract

**Objective:**

To investigate the diagnostic performance of MRI in detecting clinically significant prostate cancer (csPCa) and prostate cancer (PCa) in patients with prostate-specific antigen (PSA) levels of 4–10 ng/mL.

**Methods:**

A computerized search of PubMed, Embase, Cochrane Library, Medline, and Web of Science was conducted from inception until October 31, 2023. We included articles on the use of MRI to detect csPCa or PCa at 4–10 ng/mL PSA. The primary and secondary outcomes were MRI performance in csPCa and PCa detection, respectively; the estimates of sensitivity, specificity, positive predictive value (PPV), and negative predictive value (NPV) were pooled in a bivariate random-effects model.

**Results:**

Among the 19 studies (3879 patients), there were 10 (2205 patients) and 13 studies (2965 patients) that reported MRI for detecting csPCa or PCa, respectively. The pooled sensitivity and specificity for csPCa detection were 0.84 (95% confidence interval [CI], 0.79–0.88) and 0.76 (95%CI, 0.65–0.84), respectively, for PCa detection were 0.82 (95%CI, 0.75–0.87) and 0.74 (95%CI, 0.65–0.82), respectively. The pooled NPV for csPCa detection was 0.91 (0.87–0.93). Biparametric magnetic resonance imaging also showed a significantly higher sensitivity and specificity relative to multiparametric magnetic resonance imaging (both *p* < 0.01).

**Conclusion:**

Prostate MRI enables the detection of csPCa and PCa with satisfactory performance in the PSA gray zone. The excellent NPV for csPCa detection indicates the possibility of biopsy decision-making in patients in the PSA gray zone, but substantial heterogeneity among the included studies should be taken into account.

**Clinical relevance statement:**

Prostate MRI can be considered a reliable and satisfactory tool for detecting csPCa and PCa in patients with PSA in the “gray zone”, allowing for reducing unnecessary biopsy and optimizing the overall examination process.

**Key Points:**

- Prostate-specific antigen (PSA) is a common screening tool for prostate cancer but risks overdiagnosis.
- MRI demonstrated excellent negative predictive value for prostate cancer in the PSA gray zone.
- MRI can influence decision-making for these patients, and biparametric MRI should be further evaluated.

**Graphical Abstract:**

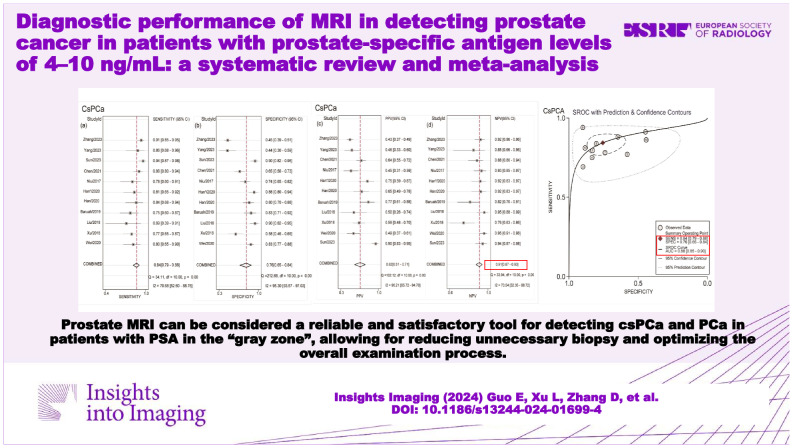

## Introduction

Prostate cancer (PCa) is one of the most frequently diagnosed cancers in men worldwide, and its prevalence continues to increase annually [1]. Thus, it is imperative to improve the accuracy of diagnosis for PCa, particularly for clinically significant PCa (csPCa) that requires curative treatment and active monitoring, so as to reduce the mortality due to malignancy [2]. As a serum marker, prostate-specific antigen (PSA) is a common clinical screening index. However, numerous trials have confirmed that this approach has the risk of over-diagnosis [3], since PSA is organ-specific but not cancer-specific. Clinically, patients with PSA levels > 10 ng/mL are highly suspected of having PCa, such that they necessitate a biopsy. In contrast, it is still debatable as to whether biopsies should be carried out in patients with PSA values in the range of 4–10 ng/mL [4], referred to as the “gray zone.” Notably, conducting biopsies in men with PSA in the “gray zone” may lead to over-diagnosis and over-treatment, as well as other negative effects, such as bleeding, genitourinary infections, and urinary retention [5].

With its morphological and various functional imaging modalities, multiparametric magnetic resonance imaging (mpMRI) of the prostate has been applied for the detection, localization, and staging of PCa, and for patient treatment planning [6]. Numerous studies have shown that using MRI prior to biopsy can diminish the detection of indolent prostate cancer while improving the accuracy of diagnosis for csPCa, thus leading to a reduction in unnecessary prostate biopsies [7, 8].

A meta-analysis published by Sathianathen et al indicated that the pooled negative predictive values (NPVs) of MRI for csPCa diagnosis with different combinations of negative mpMRI and csPCa definitions were satisfactory, ranging from 86.8 to 97.1%, suggesting that there was a reliable value for negative MRI in excluding non-csPCa patients [9]. However, this study did not analyze the efficacy of MRI in the context of the PSA gray zone, which is a challenging issue encountered in clinical practice. The diagnostic performance of prostate MRI has been widely studied recently in individuals with PSA levels of 4–10 ng/mL, albeit with high variability found among various centers.

Therefore, we conducted a comprehensive analysis of existing literature and performed a meta-analysis to investigate the performance of prostate MRI in patients with PSA levels of 4–10 ng/mL and explored the potential benefits of MRI in the management of patients in the PSA gray zone.

## Materials and methods

This meta-analysis and systematic review (CRD: 42023473553) were reported in compliance with the Preferred Reporting Items for Systematic Reviews and Meta-Analyses (PRISMA) guidelines [10].

### Search strategy and selection criteria

Two authors systematically searched PubMed, Embase, Cochrane Library, Medline, and Web of Science for studies published from inception to October 31, 2023, with language restricted to English. The search strategy is detailed in Supplemental Materials (S-1). The titles and abstracts of all studies obtained through the search strategies were independently screened by two reviewers. The reviewers then read the full text of the articles to determine whether they appropriately satisfied the inclusion criteria.

### Inclusion and exclusion criteria

Studies were considered eligible if they met the following criteria, applying the participants, intervention, control, outcomes, and study (PICOS) format.

1. P: men with elevated PSA levels in the range of 4–10 ng/mL.
2. I: patients who underwent MRI for assessing csPCa or PCa.
3. C: pathological results from radical prostatectomy or biopsy taken as the reference standard.
4. O: outcome indicators reflecting the true positive (TP), false positive (FP), false negative (FN), and true negative (TN), or the sensitivity and specificity of MRI diagnostic performance.
5. S: original articles.

The exclusion criteria included the following:

1. Articles that were unrelated to the field of interest of this study.
2. A PSA level not in the range of 4–10 ng/mL.
3. Data insufficient to construct a 2 × 2 table.
4. Non-original articles such as editorials, case reports, narrative reviews, meta-analyses, or conference abstracts.
5. Languages other than English or unavailable full text.

### Data extraction and quality assessment

The following three types of information were extracted from the included articles: (1) demographic and clinical characteristics—i.e., number of patients, number of malignancies, age, and PSA level; (2) study characteristics such as publication year, study period, country, study design, blinding, Prostate Imaging Reporting and Data System (PI-RADS) version with its cutoff value, reference standard, and analysis (patient or zone); and (3) technical characteristics of MRI, such as magnet strength, vendor, MRI sequences, number of readers, readers’ experience, and coil. The quality of the diagnostic accuracy studies was assessed by implementing the Quality Assessment of Diagnostic Accuracy Studies-2 tool (QUADAS-2) [11].

The process described above was completed by two independent reviewers (L.L.X. and E.J.G., with 6 and 2 years of experience, respectively), and disagreements were resolved through discussion or by consulting a senior reviewer (S.H.).

### Data synthesis and analysis

The heterogeneity of the results of the included studies was quantified using I^2^ statistic [12]. Cochran’s Q test with *p* < 0.1 indicated significant heterogeneity. The summary estimates of sensitivity and specificity, the combined positive predictive value (PPV) and negative predictive value (NPV), and their corresponding 95% confidence intervals (CIs) were computed using the bivariate random-effects model [13]. A hierarchical summary receiver operating characteristics (HSROC) [14] curve with a 95% confidence region and prediction region was presented graphically to illustrate our results and to show the amount of variation between the studies. The presence of publication bias was tested by applying the Deeks’ funnel plot, and statistical significance was determined by the Deeks’ asymmetry test [15].

The following categories were used in the subgroup analysis to investigate the sources of heterogeneity in the detection of csPCa.

1. PI-RADS version (PI-RADS v2.1 vs. PI-RADS v2).
2. Sequence (mpMRI vs. biparametric magnetic resonance imaging [bpMRI]).
3. Standard reference (TRUS-guided systematic biopsy [TRUS-SB] combined with cognitive MRI fusion-guided targeted biopsy [CMF-TB] vs. TRUS-SB).
4. Study design (prospective vs. retrospective).

The performance of MRI in detecting PCa was the secondary objective, with the sensitivities and specificities pooled. We then performed subgroup analysis and meta-regression based on whether or not the PI-RADS assessment was applied.

Statistical analysis was performed using Stata 14.0 (StataCorp LLC, College Station, TX, USA) with *p* < 0.05 considered the statistically significant difference. RevMan 5.3 (Cochrane Library) was implemented for processing the assessment of quality.

## Results

### Literature search

**Fig. 1:**
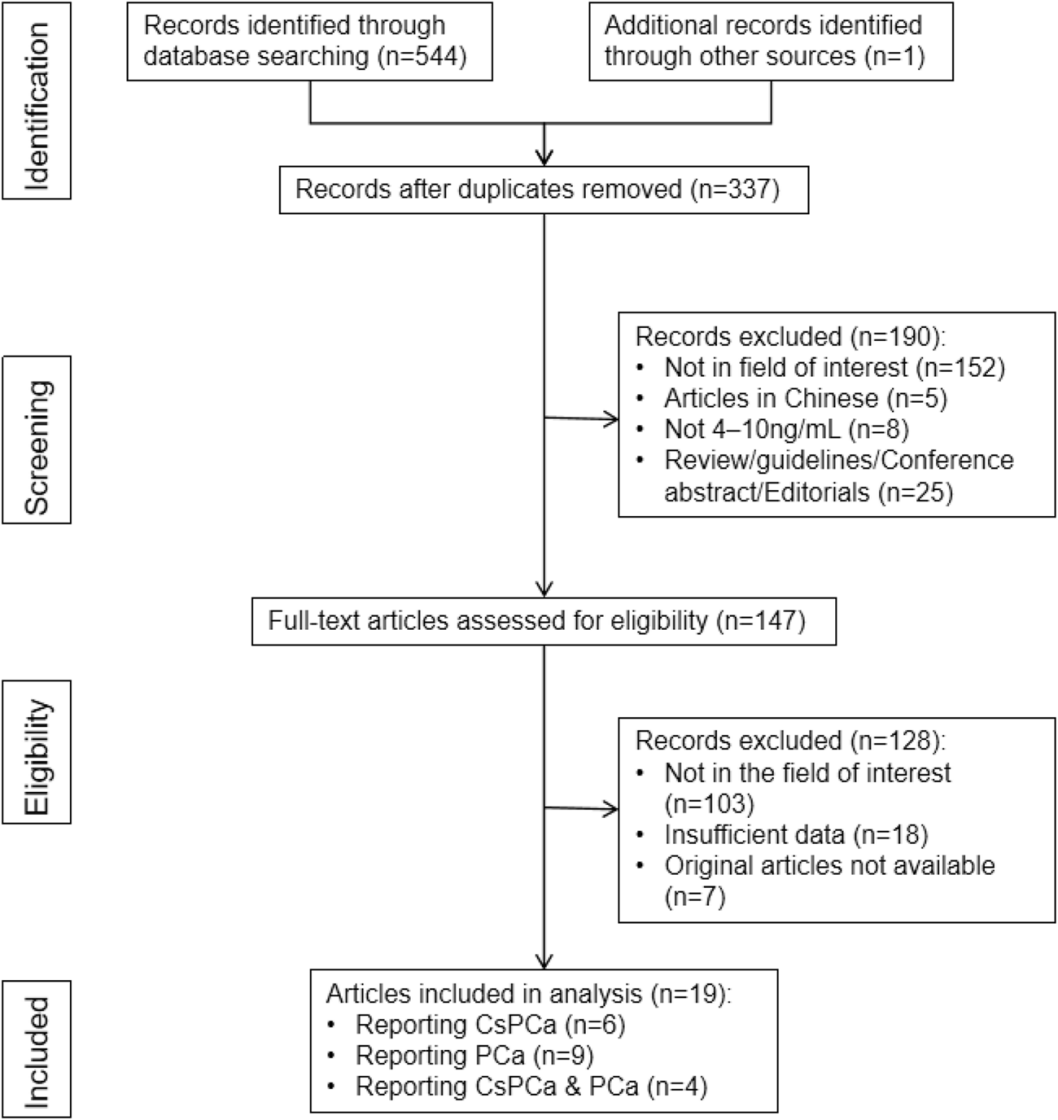
Flow diagram of the study selection process for this systematic review and meta-analysis

The detailed study selection process is presented in Fig. 1. A total of 19 studies with 3879 participants that met the inclusion criteria were chosen for the final analysis [16–34]. The authors of 10 (2205 patients) and 13 studies (2965 patients) reported the diagnostic performance of MRI for detecting csPCa [16–22, 28, 31, 32] and PCa [18, 19, 23–30, 32–34], respectively. The detailed research selection process is presented in the Supplemental Materials (S-2).

### Characteristics of the included studies

Patient characteristics are shown in Table 1, and the study characteristics are described in Table 2. The number of patients ranged from 50 to 756 patients, with a mean age of 64–74 years. Based on pathological or biopsy results, the prevalence of csPCa was calculated between 18 and 67%. The detailed characteristics of the included studies are presented in the Supplemental Materials (S-3).

**Table 1:** Patient characteristics of the included studies

First author	No. of patients	csPCa/PCa	Age (years)^a^	PSA (ng/mL)^a^
Sun [16]	200	100/NA	64.5 (60–70)	7.4 (6.0–8.5)
Wei [17]	255	44/75	71 (63–75)	7.29 (5.7–8.4)
Xu [18]	528	61/137	64 (54–82)^b^; 65 (52–82)^b^	7.50 (4.3–10.0)^b^; 6.72 (4.0–10.0)^b^
Liu [19]	102	13/15	66 (60–72)	6.91 (5.9–8.4)
Baruah [20]	104	44/NA	68 (60.3–71)	8.15 (6.4–9.7)
Han [21]	123	37/NA	66.3 ± 8.9	7.2 ± 1.5
Niu [22]	151	32/84	63.5 (65–74)	5.7 (4.8–6.7)
Qi [23]	133	NA/57	67.5 ± 7.3	7.0 ± 1.7
Tamada [24]	50	NA/35	70 (40–84)^b^	6.7 (4.1–9.9)^b^
Dwivedi [25]	137	NA/32	65.0 (65.1 ± 6 7.7)	7.2 (7.3 ± 1.7)
Vilanova [26]	52	NA/11	69 (47–87)^c^	4–10^c^
Kubota [27]	185	NA/62	68.7 ± 7.7	6.6 ± 1.7
Pepe [29]	100	NA/37	NA	8.6 (4.2–10)^b^
Sciarra [30]	90	NA/44	63.5 (49–74)^c^	6.2 ± 1.0
Chen [28]	222	94/121	67.6 ± 7.4	7.1 (5.9–8.6)
Yang [31]	81	29/NA	63.9 ± 6.5	6.9 (5.3–8.3)
Zhang [32]	439	137/186	64.9 ± 9.6	7.2 ± 1.6
Liu [33]	756	NA/160	74.3 ± 3.2	7.1 ± 1.2
Zhong [34]	171	NA/66	70.2 ± 8.4; 69.8 ± 6.5	7.7 ± 1.6; 7.4 ± 1.5

^a^ Data are presented in median (interquartile range) or mean ± standard deviation if not specified

^b^ Data are presented in the median (range)

^c^ Data are presented in mean (range)

*NA* not available

**Table 2:** Study characteristics of the included studies

First author	Period	Country	Study design	Blinded	PI-RADS version	Cutoff	Reference standard	Analysis
Sun [16]	2017.7/2021.10	China	retrospective; single center	Yes	PI-RADS v2.1 (bpMRI)	≥ 3	biopsy	patient/zone
Wei [17]	2015.1/2019.10	China	retrospective; single center	Yes	PI-RADS v2 (bpMRI)	≥ 4	biopsy	patient
Xu [18]	2015.5/2017.5	China	retrospective; single center	Yes	PI-RADS v2 (mpMRI)	≥ 4/≥ 3	biopsy	patient
Liu [19]	2015.1/2017.7	China	retrospective; single center	NA	PI-RADS v2 (mpMRI)	≥ 4/≥ 4	pathology or biopsy	lesion
Baruah [20]	2017.4/2018.10	India	prospective; single center	NA	PI-RADS v2 (mpMRI)	≥ 4	biopsy	patient
Han [21]	2010.6/2017.5	China	retrospective; single center	Yes	PI-RADS v2.1 (mpMRI and bpMRI)	≥ 4	biopsy	patient
Niu [22]	2014.1/2015.9	China	retrospective; single center	Yes	PI-RADS v2 (mpMRI)	≥ 4	pathology or biopsy	patient
Qi [23]	2015.12/2018.3	China	retrospective; single center	Yes	PI-RADS v2 (mpMRI)	≥ 4	biopsy	patient/zone
Tamada [24]	2006.1/2009.12	Japan	retrospective; single center	Yes	NA	NA	biopsy	patient/lesion
Dwivedi [25]	2009/2015	India	prospective; single center	Yes	NA	NA	biopsy	patient
Vilanova [26]	1997.1/1998.12	Spain	prospective; single center	Yes	NA	NA	biopsy	patient
Kubota [27]	2004.4/2006.3	Japan	prospective; single center	Yes	NA	NA	biopsy	patient
Pepe [29]	2011.6/2014.3	Italy	prospective; single center	Yes	NA	NA	biopsy	patient
Sciarra [30]	2007.1/2009.1	Italy	prospective; single center	NA	NA	NA	biopsy	patient
Chen [28]	2017.1/2020.1	China	prospective; single center	Yes	PI-RADS v2.1 (mpMRI)	CsPCa & PCa ≥ 4; PZ ≥ 3, TZ ≥ 4	biopsy	patient/zone
Yang [31]	2019.9/2022.1	China	retrospective; single center	Yes	PI-RADS v2.1 (mpMRI)	≥ 3	pathology	patient
Zhang [32]	2018.8/2021.7	China	retrospective; single center	NA	PI-RADS v2 (mpMRI)	≥ 4/≥ 3	biopsy	patient
Liu [33]	2014.12/2022.12	China	retrospective; single center	NA	PI-RADS (mpMRI)	≥ 3	pathology	patient
Zhong [34]	2019.1/2021.6	China	retrospective; single center	Yes	PI-RADS v2.1 (mpMRI)	NA	pathology	patient

The MRI characteristics are summarized in Table 3. Eleven studies entailed the use of 3-T scanners [17–19, 21–23, 28, 29, 31, 32, 34], six studies used 1.5-T scanners [20, 24–27, 30], one study used both [16]; one article did not provide relevant explanations [33]. The relevant elements are described in detail in the Supplemental Materials (S-4).

**Table 3:** MRI characteristics of the included studies

First author	Magnet strength (T)		Vendor	Sequences	No. of readers	Experience (years)	Coil
Sun [16]	3.0 or 1.5	Siemens; Philips; GE; UIH		T2-WI/DWI	2	10/12	The phased array coil
Wei [17]	3.0	Philips Ingenia		T2-WI/DWI	2	NA	A 32-channel body phased array coil
Xu [18]	3.0	SIEMENS VERIO		T2-WI/DWI/DCE	2	NA	NA
Liu [19]	3.0	Siemens		T2-WI/DWI/DCE	1	NA	An 18-element body coil
Baruah [20]	1.5	NA		T2-WI/DWI/DCE	NA	NA	NA
Han [21]	3.0	GE		T2-WI/DWI/DCE; T2-WI/DWI	2	≥ 5	A phased array coil
Niu [22]	3.0	Siemens		T2-WI/DWI/DCE	2	3/5	Six-channel phased-array body coil
Qi [23]	3.0	GE		T2-WI/DWI/DCE	2	1/5	NA
Tamada [24]	1.5	GE		T2-WI/DWI/DCE	2	11/7	A body coil & multichannel phased-array torso coil
Dwivedi [25]	1.5	Siemens		T2-WI/DWI/MRSI	2	21/20	An endorectal coil with pelvic phased array coil
Vilanova [26]	1.5	GE		T2-WI	2	NA	An endorectal coil
Kubota [27]	1.5	Philips		T2-WI	2	10/8	A phased array coil
Pepe [29]	3.0	Philips		T2-WI/DWI/DCE	2	NA	Surface 16 channels phased-array coil
Sciarra [30]	1.5	Siemens		MRSI/DCE	2	NA	Surface phased array and endorectal coil
Chen [28]	3.0	GE		T2-WI/DWI/DCE	2	NA	A phased-array coil
Yang [31]	3.0	Siemens		T2-WI/DWI/DCE	2	NA	An external coil
Zhang [32]	3.0	Skyra; Siemens; GE Healthcare;		T2-WI/DWI/DCE	NA	≥ 5	NA
Liu [33]	NA	NA		T2-WI/DWI/DCE	NA	NA	NA
Zhong [34]	3.0	Skyra; Siemens Healthcare		T2-WI/DWI/DCE	2	5/20	Body phased-array coil

*T2-WI* T2-weighted imaging, *DWI* diffusion-weighted imaging, *DCE* dynamic contrast-enhanced MRI, *MRSI* magnetic resonance spectroscopy imaging

### Quality assessment

In general, the quality of the studies was moderate, with 15 studies satisfying four out of seven items in the QUADAS-2 tool. The detailed quality assessment of the enrolled studies is depicted in Fig. S1, a detailed description is presented in the Supplemental Materials (S-5), and the specific evaluation results of each study are presented in Table S2.

### Diagnostic performance of MRI for detection of csPCa

**Fig. 2:**
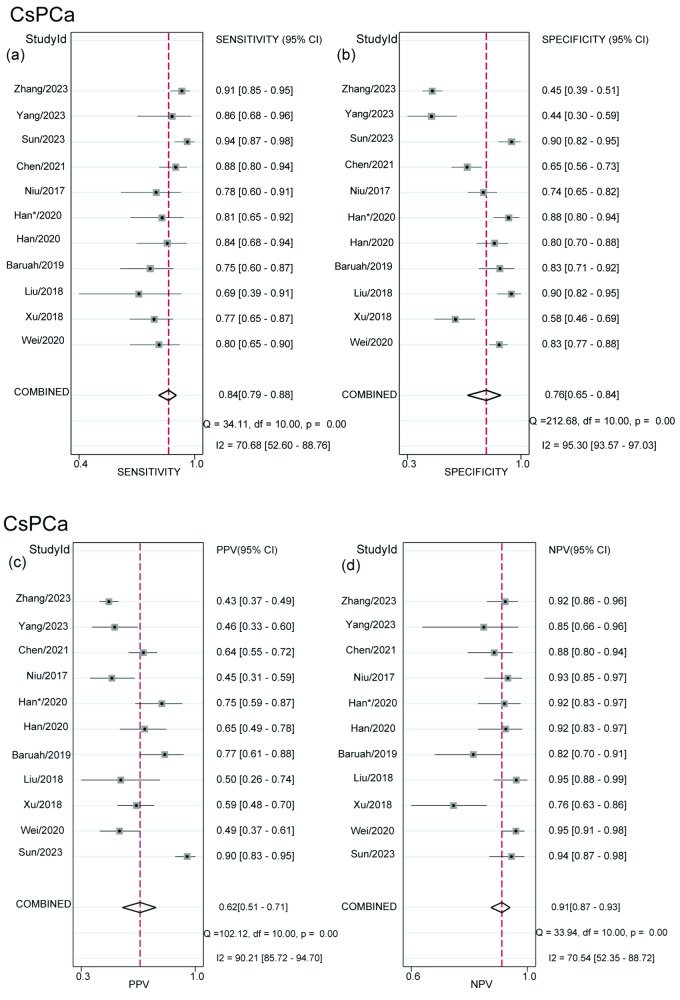
Coupled forest plot of pooled sensitivity (**a**) and specificity (**b**). Coupled forest plot of pooled PPV (**c**) and NPV (**d**). Numbers are the pooled estimates with 95%CI in parentheses. Corresponding heterogeneity statistics are provided at the bottom right corners. Horizontal lines indicate 95%CI

**Fig. 3:**
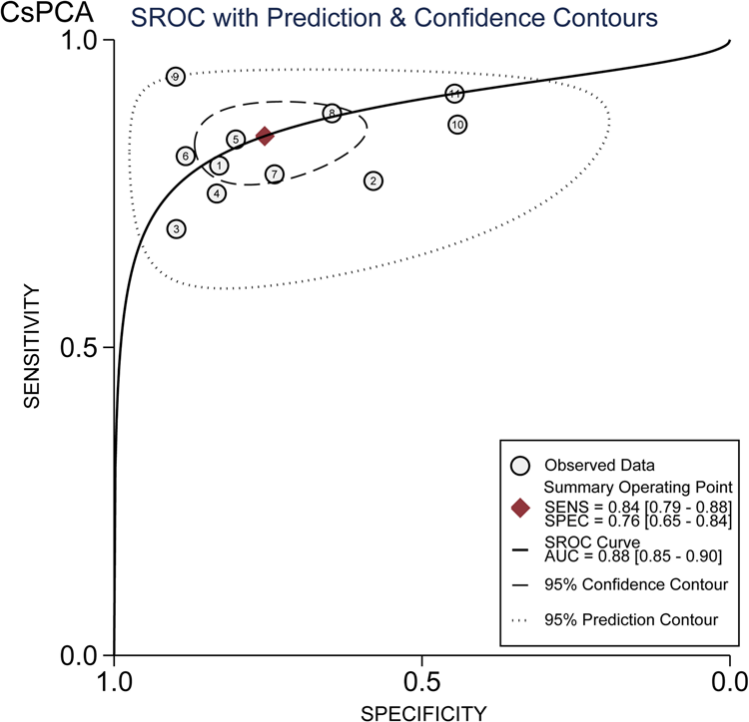
HSROC curve of diagnostic performance of MRI for csPCa detection

**Fig. 4:**
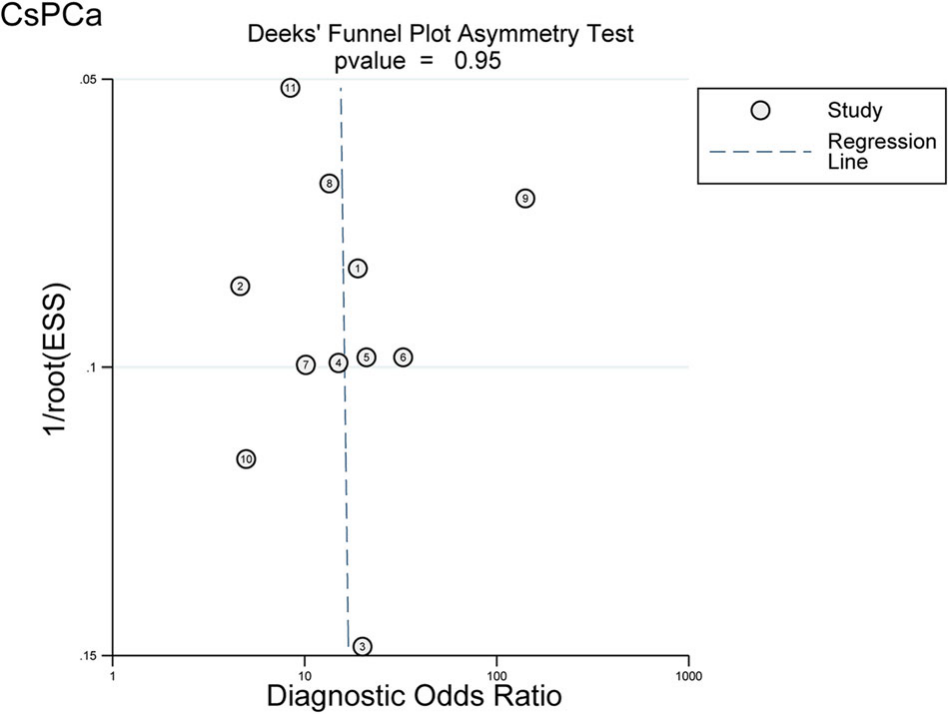
Deeks’ funnel plot. The likelihood of publication bias was low, with a *p* value of 0.95 for the slope coefficient. ESS, effective sample size

The pooled sensitivity of MRI for csPCa detection was 0.84 (95%CI, 0.79–0.88) and the pooled specificity was 0.76 (95%CI, 0.65–0.84) (Fig. 2a, b). The summary PPV and NPV were 0.62 (95%CI, 0.51–0.71) and 0.91 (95%CI, 0.87–0.93), respectively (Fig. 2c, d). The area under the HSROC curve was 0.88 (95%CI, 0.85–0.90) (Fig. 3). The Deeks’ funnel plot showed no evidence of publication bias, with a *p* value of 0.95 for the asymmetry test (Fig. 4). Heterogeneity was observed as indicated by the Cochran’s Q test (*p* < 0.01), with the *I*^2^ statistic denoting substantial heterogeneity in relation to the sensitivity (*I*^2^ = 90%) and specificity (*I*^2^ = 71%). The HSROC curve revealed significant differences between the 95% confidence and prediction zones, further highlighting the variability within the studies.

### Subgroup analysis and meta-regression

The results of the subgroup analysis and meta-regression are presented in Table 4. The pooled sensitivities and specificities were significantly different between bpMRI and mpMRI (0.87 vs. 0.83, *p* < 0.01; 0.87 vs. 0.69, *p* < 0.01). The pooled sensitivity for PI-RADS v2.1 was significantly higher than that for PI-RADS v2.0 (0.88 vs. 0.81, *p* = 0.02). As for the standard reference, TRUS-SB in combination with CMF-TB revealed significantly high sensitivity compared with TRUS-SB alone (0.88 vs. 0.77, *p* = 0.02). The pooled sensitivity of prospective studies was lower than that of retrospective studies (0.83 vs. 0.85, *p* = 0.01).

**Table 4:** Subgroup analysis of the diagnostic performance of MRI for csPCa detection

Covariate/subgroup	Studies, *n*	Sensitivity (95%CI)	*p* value	Specificity (95%CI)	*p* value
PI-RADS version			0.02		0.38
PI-RADS v2.1	5	0.88 [0.83–0.93]		0.77 [0.63–0.90]	
PI-RADS v2	6	0.81 [0.74–0.87]		0.75 [0.62–0.88]	
MRI			< 0.01		< 0.01
mpMRI	8	0.83 [0.77–0.88]		0.69 [0.59–0.80]	
bpMRI	3	0.87 [0.80–0.94]		0.87 [0.78–0.96]	
Reference standard			0.02		0.37
Cognitive MRI fusion-guided targeted biopsy	8	0.88 [0.84–0.91]		0.75 [0.64–0.86]	
TRUS-guided biopsy	3	0.77 [0.70–0.85]		0.76 [0.59–0.94]	
Design			0.01		0.58
Prospective	2	0.83 [0.73–0.93]		0.75 [0.52–0.98]	
Retrospective	9	0.85 [0.80–0.90]		0.76 [0.65–0.86]	

### Diagnostic performance of MRI for detection of PCa

The pooled sensitivity and specificity of the 13 studies [18, 19, 23–30, 32–34] for PCa detection were 0.82 (95%CI, 0.75–0.87) and 0.74 (95%CI, 0.65–0.82), respectively, with the area under the HSROC curve of 0.85 (95%CI, 0.82–0.88) (Figs. S2 and S3). Detailed information on secondary outcomes is depicted in the Supplemental Materials (S-6). We further conducted subgroup analysis on the diagnostic performance of MRI in the diagnosis of prostate cancer according to whether PI-RADS was used. The results are provided in Table S1.

## Discussion

In this meta-analysis, we investigated the diagnostic efficacy of MRI in the detection of both csPCa and PCa among patients with PSA levels between 4 and 10 ng/mL. Generally, MRI demonstrated a favorable diagnostic performance for csPCa detection, with the area under the HSROC curve, sensitivity, and specificity of 0.88 (0.85–0.90), 0.84 (0.79–0.88), and 0.76 (0.65–0.84), respectively. The pooled NPV of prostate MRI for csPCa detection was satisfactory with the value of 0.91 (0.87–0.93). Regarding PCa detection, the pooled sensitivity and specificity were 0.82 (0.75–0.87) and 0.74 (0.65–0.82), respectively.

The pooled NPV for csPCa detection obtained in our study was excellent, indicating that there is sufficient certainty to exclude non-csPCa patients when the MRI result is negative [9], which would lead to a reduction in unnecessary biopsies. A previous study shown that in patients with a PSA < 10 ng/mL, the median NPV of MRI for overall PCa was 86.3% (IQR, 73.3%–93.6%), with a median cancer prevalence of 35.4% (IQR, 27.6–42.5%) [35]; this was similar to our research results. Similarly, the results of the study published by Xu et al showed that at a median PSA value of 4.65 (0.22–86.00) ng/mL and a csPCa prevalence of 42%, the NPV of mpMRI for csPCa detection was 87.8%, whereas the NPV of bpMRI for csPCa detection was 85.0% [36]. Although the NPV varied in the studies included in our analysis (ranging from 76–95%—potentially the result of the heterogeneous prevalence among them, which ranged between 18 and 67%), this indicator was generally satisfactory and supported the benefits of MRI in reducing unnecessary biopsies.

Compared with the excellent pooled NPV in our study, the pooled PPV of 0.66 (0.54–0.76) was less than ideal. Because prostate MRI is a screening tool and clinical priority is defined as not missing any significant cancer, we paid more attention to the NPV of MRI screening. However, the PPV also reflects vital clinical significance as it describes whether positive mpMRI consistently supports the presence of csPCa. Regarding the relatively low PPV for patients with a positive prostate MRI, additional clinical information may need to be considered before proceeding to a biopsy. A review by Schoots et al argued that multivariable risk prediction tools—including clinical, biochemical parameters and MRI suspicion scores—possess the potential to significantly reduce the number of biopsies and the detection of clinically insignificant prostate cancer; these tools will then assist doctors and patients in making appropriate biopsy decisions [37]. Among these modalities, PSA density (PSAD) is an important parameter in guiding biopsy decisions. In a systematic review by Wang et al, a quantitative risk assessment was performed that combined different PSAD cut-offs and MRI results to predict the occurrence of csPCa [38]. PSAD demonstrated complementary performance and predictive value, especially among patients with negative MRI and PI-RADS 3 or Likert 3 lesions. However, the diagnostic performance of bp-MRI combined with PSAD did not demonstrate statistically significant improvement in all evaluation schemes, according to the research by Cuocolo et al [39]. In light of these findings, the role of PSAD remains to be further investigated. We envision that incorporating prostate MRI, clinical factors, and possible biomarkers into the biopsy decision-making process will enhance the diagnostic accuracy and increase the confidence to avoid unnecessary biopsies in patients with PSA gray zone.

Considerable heterogeneity was observed among the included studies regarding the PI-RADS versions, standard reference, and study design. Compared with three studies that entailed bpMRI, eight studies with mpMRI produced relatively low pooled sensitivity and specificity. In consensus with our results, in the study by Han et al [21], the area under the curve value for bpMRI in csPCa detection (0.86) was significantly higher than that for mpMRI (0.82). In addition, several published meta-analyses in which the diagnostic effectiveness of bpMRI and mpMRI were compared, suggested that bpMRI exhibited performance comparable to that of mpMRI when diagnosing csPCa in men with any PSA level [36, 40]. As a standard acquisition protocol of mpMRI, DCE images are of limited value in prostate cancer detection according to the PI-RADS recommendations. A study reported by Messina et al [41] suggested that upgrading peripheral lesions with DWI Score 3 to PI-RADS 4 due to a positive DCE negatively impacted the accuracy of MRI and decrease the true csPCa detection rate of PI-RADS 4 lesions. Nevertheless, DCE remains valuable in cases when DWI and/or T2WI do not reach an adequate level of quality. The ESUR/ESUI expert panel has emphasized the importance of regularly monitoring and reporting MRI quality in clinical practice [42]. The PI-QUAL scoring system is a useful tool for standardized quality assessment and reporting, with a potential impact on patient care [43]. Ponsiglione et al found that the detection efficiency of extracapsular extension was significantly improved in high-quality mpMRI scans, with diagnostic accuracy improving from 0.564 in low-quality scans to 0.849 in high-quality scans (PI-QUAL ≥ 4) [44]. A study by Brembilla et al showed that in scans of suboptimal quality, the proportion of biopsies for PI-RADS 3 MRI rose by 18% while the detection rate of csPCa declined by 35%, confirming the potential impact of MRI scan quality on the performance of mpMRI relative to biopsy results [45].

The inherent disadvantages of setting bpMRI as a default approach should also be noted, including low reproducibility in community hospitals, lack of widely accepted imaging quality standards, and the impossibility of performing loco-regional staging [46]. However, early detection of csPCa by MRI is a priority. Given the analysis above, we postulate that bpMRI could serve as a potential substitute for mpMRI to optimize the clinical workup in men with PSA levels of 4–10 ng/mL, allowing us to avoid the extra expense and scan time as well as the side effects of contrast media. Moreover, there is an urgent need for the standardization of prostate bpMRI acquisition and reporting, and more robust validations of this imaging methodology should be carried out [47].

An additional significant factor that affected heterogeneity in the sensitivity was standard reference. Eight studies using TRUS-SB combined with CMF-TB as a standard reference produced significantly higher sensitivity than the other three studies that did not incorporate CMF-TB. A comparative study conducted by Elkhoury et al [48] revealed that the clinically significant prostate cancer detection rate (CDR) by systematic biopsy was 15.7%, while the CDR using cognitive fusion biopsy was 33.3% on a per-core basis. The reason behind our outcome may have been the greater CDR of CMF-TB. CMF-TB involves an operator who is cognitively aware of the obtained MRI interpretation and uses anatomic landmarks to target suspicious lesions on real-time transrectal ultrasound (TRUS) [49]. There has thus been an improvement in the diagnostic performance of prostate MRI commensurate with the increasing rate of cancer detection.

Li et al in a relevant review systematically evaluated the effectiveness of MRI and magnetic resonance spectroscopy (MRS) in detecting PCa and csPCa in patients with PSA levels in the gray zone before biopsy, as well as their applications in guiding prostate biopsy [50]. As one of the sequences of mpMRI, MRS imaging (MRSI) enables noninvasive assessment of certain metabolites in the prostate gland; however, it is not recommended in the latest version of PI-RADS. Thus, we did not include articles about MRS. In addition, our study is an adjunct to clinical practice, not only summarizing the diagnostic efficacies of prostate MRI for csPCa in patients in the PSA gray zone, but also further assessing the impact of prostate MRI on the decision-making of patients with PSA gray zone.

Our study has several limitations. First, the design of the majority of studies regarding csPCa detection was retrospective, which may have generated some bias in the patient selection domain. Second, there was significant heterogeneity among the studies, thus affecting the general applicability of our summary estimates. We conducted subgroup analyses and meta-regression to explore the potential factors underlying the heterogeneity, but a portion of heterogeneity remained unexplained. Finally, even though our study showed that MRI screening for csPCa detection in men with PSA levels of 4–10 ng/mL exhibited satisfactory diagnostic performance with good NPV and moderate PPV, the prevalence is an influencing factor that should be taken into consideration when conducting clinical decision strategies.

## Conclusion

In this study, it was found that MRI could be considered a reliable and satisfactory tool to instruct clinical decisions for patients with PSA in the “gray zone,” particularly for csPCa detection. Furthermore, the high NPV of prostate MRI for csPCa detection indicates that negative MRI can reliably rule out the non-csPCa, sparing patients unnecessary biopsy.

### Supplementary information

ELECTRONIC SUPPLEMENTARY MATERIAL

## Data Availability

Information on study subjects was based on the previously published articles which were included in this study.

## Abbreviations

bpMRI: Biparametric magnetic resonance imaging
CI: Confidence interval
CMF-TB: Cognitive MRI fusion-guided targeted biopsy
csPCa: Clinically significant prostate cancer
HSROC: Hierarchical summary receiver operating characteristics
mpMRI: Multiparametric magnetic resonance imaging
MRS: Magnetic resonance spectroscopy
NPV: Negative predictive value
PCa: Prostate cancer
PI-RADS: Prostate Imaging Reporting and Data System
PPV: Positive predictive value
PSA: Prostate-specific antigen
PSAD: Prostate-specific antigen density
QUADAS–2: Quality Assessment of Diagnostic Accuracy Studies–2
TRUS-SB: Transrectal ultrasound-guided systematic biopsy
