# Supplementary material for: Diagnostic performance of MRI in detecting prostate cancer in patients with prostate-specific antigen levels of 4–10 ng/mL: a systematic review and meta-analysis
Source: Insights Imaging. 2024 Jun 18;15:147. doi: 10.1186/s13244-024-01699-4 (PMC11183000; doi:10.1186/s13244-024-01699-4)
Supplement: Supplementary file 1 — ELECTRONIC SUPPLEMENTARY MATERIAL [file 13244_2024_1699_MOESM1_ESM.pdf]

Diagnostic performance of MRI in detecting prostate cancer in patients with prostate-specific antigen levels of 4–10 ng/mL: a systematic review and meta-analysis

ELECTRONIC SUPPLEMENTARY MATERIAL

S-1. Search strategy

The search strategy was as follows: ([MR] or [MRI] or [multiparametric MRI] or [biparametric MRI] or [magnetic resonance imaging] or [magnetic resonance] or [PI-RADS]) and ([prostate cancer] or [PCa] or [prostate carcinoma] or [prostatic neoplasms] or [prostatic cancer]) and ([prostate-specific antigen] or [PSA]) and ([gray zone] or [4–10 ng/mL] or [4 and 10 ng/mL] or [4–10]).

S-2. Research selection process

Initially, our literature search yielded 545 results, of which 208 were removed because of the presence of duplicates. After screening the 337 titles and abstracts, 190 studies were excluded. When we performed full-text analysis of the remaining 147 potentially eligible articles, 128 studies were excluded for the following reasons: articles that were not in the field of interest of this study (n = 103); there were articles without sufficient data needed to reconstruct 2 × 2 tables (n = 18)， or the original articles were not available (n = 7). Finally, 19 studies with 3,879 participants that met the inclusion criteria were chosen for the final analysis[16-34]. Of these, 10 and 13 studies reported the diagnostic performance of MRI for detecting csPCa[16-22, 28, 31, 32] and PCa[18, 19, 23-30, 32-34], respectively.

S-3. Characteristics of included studies

The number of patients ranged from 50 to 756 patients, with a mean age of 64–74 years. Based on

pathological or biopsy results, the malignant rate was calculated from 18 to 67%. The PSA levels of participants ranged from 4 to 10 ng/mL.

In terms of PI-RADS version, PI-RADS v2.1 was utilized in five studies[16, 21, 28, 31, 34] and PI-RADS v2 was reported in seven studies[17-20, 22, 23, 32], with corresponding cut-off values for defining csPCa or PCa stated for both versions; however, the methodology was unclear in the other seven studies[24-27, 29, 30, 33]. Seven studies were prospective in terms of study design[20, 25-30], whereas the other 12 studies were retrospective[16-19, 21-24, 31-34]. Most of the studies explicitly stated the interpretation of MRI using the blinded method, whereas five studies did not clearly report this[19, 20, 30, 32, 33]. Only one study was based on lesions analysis[19].

S-4. MRI characteristics

The authors of 11 studies adopted 3-T scanners [17-19, 21-23, 28, 29, 31, 32, 34], six studies used 1.5-T scanners[20, 24-27, 30], one study used both[16], while an article did not provide relevant explanations[33]. In 13 studies, mpMRI (T2WI, DWI, DCE) was used for detecting cancer[18-24, 28, 29, 31-34], and bpMRI (T2WI, DWI) was utilized in four studies[16, 17, 21, 25]. The authors of two studies only described using T2WI sequences[26, 27], and in one study, only DCE sequences were used to scan the patients[30]. Seven of the 10 included studies entailed the application of transrectal ultrasound-guided systematic biopsy (TRUS-SB) combined with CMF-TB[16, 19, 21, 22, 28, 31, 32], while the authors of three studies did not use CMF-TB[17, 18, 20].

S-5. Quality Assessment (QUADAS-2 tool)

Regarding the patient selection domain, two studies exhibited a high risk of bias, as they were case-

control studies in design[16, 33]. Additionally, four studies[17, 18, 24, 29] in which the authors did not report whether or not patients were enrolled consecutively, had an “unclear” score. Two studies had a “high” concern of applicability because they exclusively included patients with first negative biopsy[29, 30]. Concerning the index test domain, there was a high risk of bias in one study as that its threshold for defining csPCa and CA was not prespecified[28]. And because five studies did not specify whether the MRI was read while being blinded to clinical-pathological information, we designated them as reflecting an unclear risk of bias[19, 20, 30, 32, 33]. Regarding the reference standard domain, there was a high risk of bias in 12 studies since the pathologists were aware of the MRI interpretation[16, 19, 22, 23, 26, 28, 29, 31, 32, 34]. Concerning the flow and timing domain, one study was given a high risk of bias since it used both pathological specimens after RP and biopsy as standard references for adapting to different patient situations[19].

S-6. Diagnostic performance of MRI for detection of PCa

The I^2 statistic demonstrated substantial heterogeneity for predicting PCa, and the Cochran’s Q test provided evidence of heterogeneity ($p<0.01$). The pooled sensitivity and specificity of the 13 studies[18, 19, 23-30, 32-34] were 0.82 (95% CI, 0.75–0.87) and 0.74 (95% CI, 0.65–0.82), respectively, with the area under the HSROC curve of 0.85 (95% CI, 0.82–0.88) (Figure S2 and S3). The Deeks’ funnel plot and asymmetry test revealed that there was no significant likelihood of publication bias ($p=0.17$; Figure S4). The subgroup analyses and meta-regression showed that the use PI-RADS was a significant factor accounting for the heterogeneity on the sensitivity, with 0.86 (95% CI, 0.81–0.92) for using PI-RADS and 0.74 (95% CI, 0.64–0.85) for not using PI-RADS. However, there was no difference in specificity between the two subgroups (0.76 [95% CI, 0.66–0.86] vs. 0.71 (95% CI, 0.58–0.84], $p=0.09$) (Table S1).

Table S1. Subgroup analysis of the diagnostic performance of MRI for PCa detection.

Covariate/Subgroup	Studies, n	Sensitivity (95% CI)	p-value	Specificity (95% CI)	p-value
PI-RADS	7	0.86 (0.81 - 0.92)	<0.01	0.76 (0.66 - 0.86)	0.09
Non-PI-RADS	6	0.74 (0.64 - 0.85)		0.71 (0.58 - 0.84)	

Note—PI-RADS, Prostate Imaging Reporting and Data System; CI, confidence interval.

Table S2. The specific evaluation results of each study according to the QUADAS-2

Domains / Studies	PATIENT SELECTION				INDEX TEST(S)			REFERENCESTANDARD			FLow AND TIMING			
	Was a consecutive or random sample of patients enrolled?	Was a case-control design avoided?	Did the study avoid inappropriate exclusions?	Is there concern that the included patients do not match the review question?	Were the index test results interpreted without knowledge of the results of the reference standard?	If a threshold was used, was it pre-specified?	Is there concern that the index test, its conduct, or interpretation differ from the review question?	Is the reference standard likely to correctly classify the target condition?	Were the reference standard results interpreted without knowledge of the results of the index test?	Is there concern that the target condition as defined by the reference standard does not match the review question?	Was there an appropriate interval between index test(s) and reference standard?	Did all patients receive a reference standard?	Did patients receive the same reference standard?	Were all patients included in the analysis?
Baruah 2019	Y	Y	Y	Low	U	Y	Low	Y	Y	Low	Y	Y	Y	Y
Chen 2021	Y	Y	Y	Low	Y	N	Low	Y	N	Low	U	Y	Y	Y
Dwivedi 2018	Y	Y	Y	Low	Y	Y	Low	Y	N	Low	Y	Y	Y	Y
Han 2020	Y	Y	Y	Low	Y	Y	Low	Y	N	Low	Y	Y	Y	Y
Kubota 2008	Y	Y	Y	Low	Y	Y	Low	Y	Y	Low	U	Y	Y	Y
Liu 2018	Y	Y	U	Low	U	Y	Low	Y	N	Low	U	N	N	Y
Niu 2017	Y	Y	U	Low	Y	Y	Low	Y	N	Low	U	Y	Y	U
Pepe 2015	U	Y	U	High	Y	Y	Low	Y	N	Low	Y	Y	Y	Y
Qi 2020	Y	Y	U	Low	Y	Y	Low	Y	N	Low	Y	Y	Y	Y
Sciarra 2010	Y	Y	Y	High	U	Y	Low	Y	Y	Low	Y	Y	Y	Y
Sun 2023	Y	N	N	Low	Y	Y	Low	Y	N	Low	Y	Y	Y	Y
Tamada 2011	U	Y	U	Low	Y	Y	Low	Y	Y	Low	Y	Y	Y	Y
Vilanova 2001	Y	Y	Y	Low	Y	Y	Low	Y	N	Low	Y	Y	Y	Y
Wei 2020	U	Y	U	Low	Y	Y	Low	Y	Y	Low	U	Y	Y	Y
Xu 2018	U	Y	U	Low	Y	Y	Low	Y	Y	Low	Y	Y	Y	Y
Yang 2023	Y	Y	Y	Low	Y	Y	Low	Y	N	Low	Y	Y	Y	Y
Zhang 2023	Y	Y	Y	Low	U	Y	Low	Y	N	Low	U	N	N	Y
Liu 2023	Y	N	N	High	U	Y	Low	Y	U	Low	U	Y	Y	Y
Zhong 2023	Y	Y	Y	Low	Y	Y	Low	Y	N	Low	Y	Y	Y	Y

Note—Y, Yes; N, No; U, Unclear.

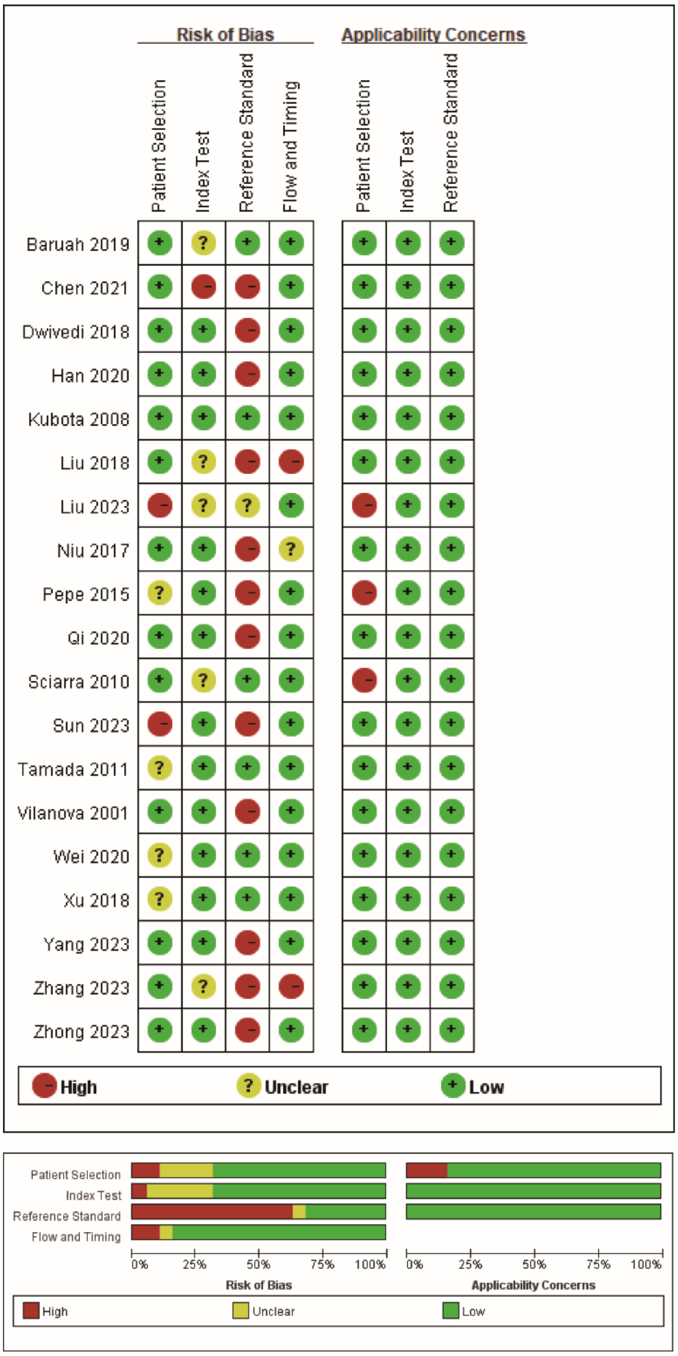

Figure S1. Risk of bias and concerns of applicability for 19 studies using the QUADAS-2 tool.

QUADAS-2, Quality Assessment of Diagnostic Accuracy Studies-2

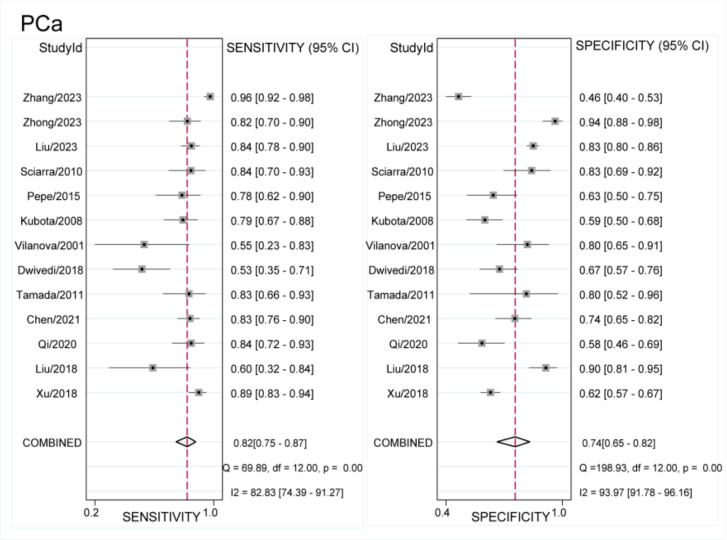

Figure S2. Coupled forest plot of pooled sensitivity and specificity. Numbers are pooled estimates with 95% CI in parentheses. Corresponding heterogeneity statistics are provided at bottom right corners. Horizontal lines indicate 95% confidence intervals (CI).

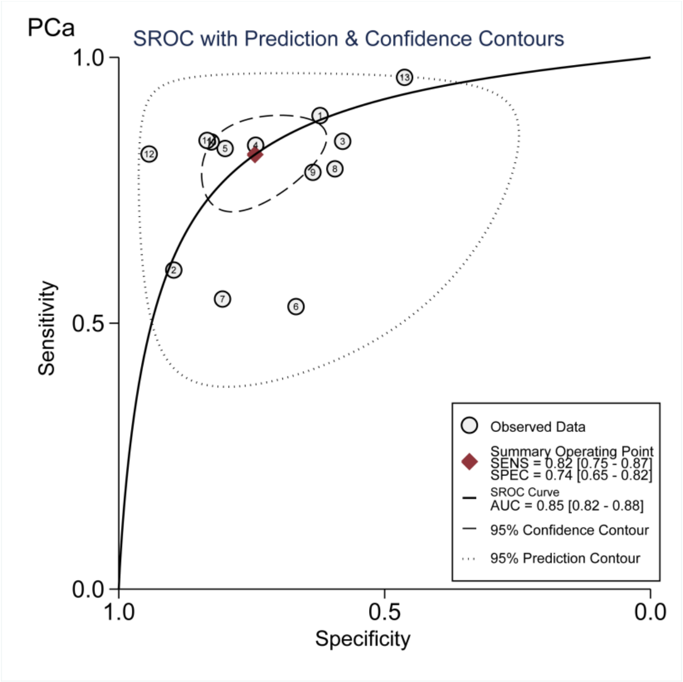

Figure S3. HSROC curve of diagnostic performance of MRI for PCa detection. HSROC = hierarchical summary receiver operating characteristic

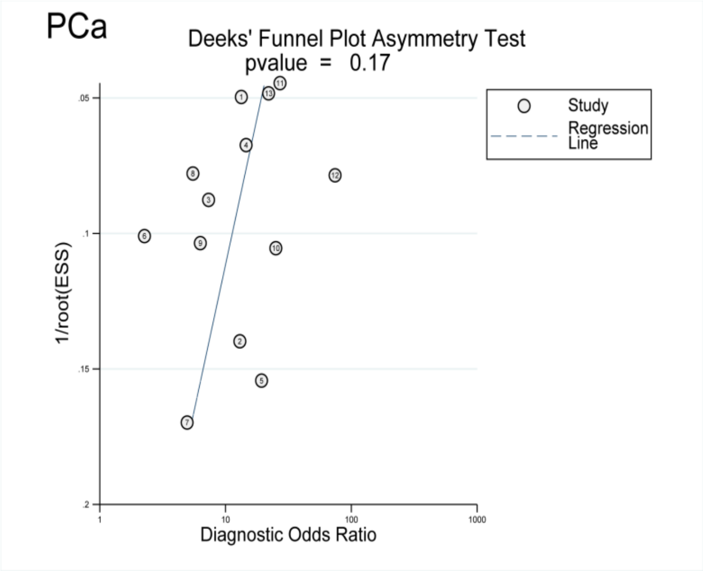

Figure S4. Deeks' funnel plot. Likelihood of publication bias was low with a p-value of 0.17 for the slope coefficient. ESS, effective sample size
